# Method Effects and the Meaning of Measurement

**DOI:** 10.3389/fpsyg.2013.00169

**Published:** 2013-04-15

**Authors:** Andrew Maul

**Affiliations:** ^1^Research and Evaluation Methodology, School of Education, University of Colorado at BoulderBoulder, CO, USA

**Keywords:** method effects, multi-trait multi-method, measurement theory, scientific realism, semantics, generalized latent variable models

## Abstract

Although the idea of a *method effect* in psychological measurement seems intuitively straightforward – that is, it is said to occur when any characteristic of a measurement procedure contributes variance to scores beyond what is attributable to variance in the attribute of interest – much of the surrounding conceptual vocabulary remains confused. In part, these confusions can be traced to deeper confusion in the human science literature regarding the meaning of *measurement*. In particular, the thinking of human scientists about method effects has been shaped by (a) received wisdom regarding why method effects are problematic to begin with, and, therefore, what corrective measures are appropriate, (b) the formal and implied semantics of psychometric techniques that have been developed to model method effects, and (c) general philosophical undercurrents that have contributed to the collective understanding of psychological measurement. Notably, tensions between lines of thought that can be broadly characterized as *empiricist* and *realist* have contributed to uneven thinking surrounding the concept of a method effect. In this paper, it is argued that it may be possible to formulate an account of what method effects are that is coherent not only across different research traditions in the human sciences, but also with thinking found in other scientific disciplines; however, doing so requires a more explicit commitment to a realist position on measurement than is generally forthcoming from human scientists. By examining these issues, this paper hopes to contribute to semantic clarity regarding not just method effects, but also the meaning of measurement in psychology.

Although the ideas of *method effects* and *methods variance* have been present in the literature on psychological measurement for more than a half-century, and at first glance seem to be intuitively straightforward, a great deal of the associated conceptual vocabulary remains confused. In part these confusions can be traced to the significant variation in the ways in which method effects are conceptualized, discussed, and statistically modeled in different parts of the literature; in particular, discourse on method effects is shaped by the explanations given for why method effects are problematic to begin with, and by the semantics of commonly applied statistical models for behavioral data [particularly confirmatory linear factor analysis (CFA) and fixed- and random-effects perspectives on the modeling of local item dependence (LID) within the item response theory (IRT) tradition]. Additionally, and more fundamentally, confusions regarding method effects are connected to deeper confusions regarding the meaning of *measurement* in the human sciences.

Method effects are generally understood as occurring when “any characteristic of a measurement process or instrument contributes variance to scores beyond what is attributable to the construct of interest” (e.g., Sechrest et al., 2000, p. 64). It is regularly pointed out, however, that this definition lacks rigor, and thus there is considerable variation in interpretations of the concept (e.g., Fiske and Pearson, 1970; Golding, 1977; Fiske and Campbell, 1992; Cronbach, 1995; Brannick et al., 2010). To begin with, the definition of a method effect in terms of sources of variance in outcomes seems to be both too strong and too weak: too strong, as it would exclude systematic biasing effects that would leave score variance intact (i.e., effects that operate on the expectation of the outcome of a procedure rather than its variance), and too weak, as it would include anything that adds random noise to the outcomes of a procedure, which would seem to imply that unreliability in general is a kind of method effect. Further, most of the time method effects are defined by ostension, and the examples commonly given can vary significantly from one field to another. Consequentially, the operating definition of a method effect used by most applied researchers is loose and relative to the purposes of a test (Sechrest et al., 2000), and may or may not include such things as person factors (e.g., response biases such as halo effects and social desirability; Bagozzi and Yi, 1991) and situational factors (e.g., whether a test is administered in a high-stakes context). Additionally, standard thinking about method effects is based on a strict trait-method dichotomy that is itself questionable (Cronbach, 1995), and may be more applicable in some contexts than others. Even researchers within the same field may have very different understandings of what a method effect is and how much of a problem it is (Brannick et al., 2010; Pace, 2010).

Furthermore, the term “method effect” is itself only commonly encountered in a particular subset of human science fields of research, such as those concerned with personality, organizations, management, and marketing. The concept of shared variance among behavioral observations due to communality in incidental features of the observational procedure is present in other fields as well, though it is often couched in different terms, such as measurement invariance to transformations of test features, generalizability across different response formats or modes of observation, and item-type-related LID. Accompanying these distinct vocabularies are distinct statistical tools, and distinct motivations for concern with method effects. These differences, combined with the simple fact that the models and their applications appear in research journals with little overlap in readership, has led to a significant degree of unevenness in thinking about the role of methods in measurement throughout the human sciences.

An additional and more fundamental factor contributing to confusion regarding method effects is that discourse and thinking on method effects in measurement depends on the way in which measurement itself is conceived. Especially in recent years, a number of scholars have noted a great deal of confused thinking in the human sciences regarding the meaning of measurement (e.g., Michell, 1990, 1997, 1999, 2000, 2004, 2005, 2008; Borsboom et al., 2003, 2004; Borsboom, 2005, 2008; Trendler, 2009; Sijtsma, 2012). In part this confusion is due to the fact that two general currents of philosophical thought in the twentieth and twenty-first centuries have influenced thinking about psychological measurement, in some cases in conflicting ways. In particular, while *empiricism* (and, more particularly, logical positivism, and operationalism) strongly influenced the early development of theories of measurement in psychology (e.g., Boring, 1923; Bridgman, 1927; Boring et al., 1945; Stevens, 1946; Suppes and Zinnes, 1963; Krantz et al., 1971), philosophical *realism* has arguably underpinned many of the uses and interpretations of psychological tests from the beginning (a point argued by Michell, 1999), and has been more explicitly invoked in theories of measurement and validity in recent years (e.g., Michell, 1990, 1999, 2005; Borsboom et al., 2004; Borsboom, 2005; Hood, 2009; Maul, 2012; also in the “constructive-realism” of scholars such as Messick, 1989, and Mislevy, 2009; see also Slaney and Racine, 2011). The average practitioner or researcher may be unaware of the conceptual tensions that sometimes result from the different ways in which these two strands of thought have contributed to discourse about specific concepts such as method effects, and about measurement more generally.

It may be possible to formulate an account of what method effects are that is coherent not only across different research traditions in the social sciences, but also with thinking found in other scientific disciplines; however, doing so requires a more explicit commitment to a realist position on measurement than is generally forthcoming from human scientists. By examining these issues, this paper hopes to contribute to semantic clarity regarding not just method effects, but also the meaning of measurement in psychology. The organization is the paper is as follows. The first section reviews the logic commonly given in different parts of the literature about what method effects are and why they are important. The second section more closely examines the semantics of two classes of statistical tools commonly used to model method effects, and considers their implied stances on what method effects – and, by implication, measurement – are all about. The third section then delves more deeply into the ways in which general lines of philosophical thought have influenced thinking about measurement in psychology, and method effects in particular. The final section argues that, despite differences in vocabulary and norms of practice across fields, there is a common general understanding of what method effects are, and that this understanding is consistent with modern realist thinking about measurement.

## What are Method Effects, and Why are They Important?

The general understanding of method effects seems to be that they represent unwanted “nuisance” variance in observed outcomes that are associated with the particular way information was collected, rather than with variance in that which was intended to be measured (often an attribute of a person). This implies that, at least in principle, it would have been possible to collect information about the same attribute using a different method – that is, at least some features of the particular method or methods employed are incidental rather than essential features of the testing procedure. (This, as it happens, it not always obviously true; this point will be revisited in a later section).

In some cases, alternative methods may be explicitly included as design elements in a testing situation. Seen this way, the use of different methods to assess a trait represents an attempt at triangulation, or, alternatively put, an attempt to establish that inferences about a targeted attribute are robust (i.e., invariant) to a specific set of transformations in the testing procedure. For example, if information about an employe’s conflict-resolution ability is collected via self-report, peer-report, and supervisor-report, the three methods are thought to represent three different sources of evidence about the same person attribute: each of them may be flawed in particular ways, but, ideally, they are differently flawed, and so the information obtained from the three methods together may be more dependable and generalizable than the information obtained from each method individually.

Campbell and Fiske (1959) gave a related but slightly different motivation for the deliberate inclusion of different methods in their seminal article on multi-trait multi-method (MTMM) studies. Campbell and Fiske described the logic of convergent and discriminant validity,^1^ which leverages the idea of a *trait* as “a relatively stable characteristic of a person – an attribute, enduring process, or disposition – which is constantly manifested to some degree when relevant, despite considerable variation in the range of settings and circumstances (of observation)” (Messick, 1989, p. 15). If such a trait has truly been identified, and is indeed invariant to a variety of transformations in the incidentals of its assessment, different methods of observation should give consistent results. Conversely, when a particular method of observation is applied to the assessment of distinct traits, the fact that they share a method in common should not inflate their apparent association. Thus convergent validity is seen as the extent to which different methods agree on the trait values of individuals, and discriminant validity is the extent to which different traits are empirically distinguishable, even when they are assessed via the same method.

Clearly, the second of these concerns is unique to measurement situations in which more than one attribute is of interest (i.e., “multi-trait” studies). Such situations are more common in some fields, such as personality, organizational, management, and marketing research, than in others, such as educational testing and experimental psychology. Within the former collection of fields, the potential biasing of observed associations among theoretically distinct traits seems to be thought of as the primary reason why one should be concerned with method effects (e.g., Podsakoff et al., 2003; Lance et al., 2010; Pace, 2010), sometimes to the point that it is argued that method effects can be ignored entirely if this particular concern can be dismissed.

Conversely, in fields in which it is more the norm of practice to focus on the measurement of a single attribute at a time, the term “method effect” itself is less commonly heard, but the concept of variance in behavioral observations being related to incidental features of the observational procedure is nonetheless present. For example, in situations in which multiple raters score performances of individuals, the specific rater or raters to which one was assigned is generally considered an incidental, rather than essential, feature of the testing procedure. Though steps are usually taken to maximize the interchangeability of raters (e.g., by training them against a standardized rubric until a high degree of consistency is achieved), it is nevertheless well-known that even well-trained raters may introduce some amount of nuisance variance into scores due to their idiosyncrasies. Discussion of rater effects often takes place under the umbrella concept of *facets* of a testing procedure (e.g., Cronbach et al., 1972; Linacre, 1989), which could also include such things as contextual factors (e.g., whether a person is tested at morning or at night, or in a quiet environment or a noisy one), item formats or modalities (e.g., whether an item is multiple-choice, written-response, or answered orally), and delivery methods (e.g., whether a test is presented via paper and pencil or via computer). Within the context of generalizability theory (Cronbach et al., 1972), the reason for concern with facets is generally worded in terms of minimizing unwanted sources of variance when making inferences and decisions about individuals. In some other contexts, such as in many-facets Rasch measurement (Linacre, 1989), the concern is sometimes worded in terms of accounting for the fixed effects of facets on item difficulty (e.g., rater leniency or severity) and for obtaining scale scores that control for variation in construct-irrelevant facets (e.g., the specific rater or raters to which a student was assigned).

The concept of *LID*, commonly encountered in the literature on IRT, is also related to the concept of a method effect. Specifically, on a unidimensional test^2^, LID is said to occur when responses to individual items share more in common with one another than just that their probabilities jointly depend on the latent variable. This can occur for many reasons; one common example is the “testlet” situation (e.g., Wainer et al., 2007), in which specific groups of test items share, in addition to dependence on the latent variable, incidental test features such as common stimulus material, a common prompt or item stem, or a common format. The main concern that is usually given regarding LID is that failing to adequately model it will lead to overestimation of measurement precision (reflected in, for example, upwardly biased reliability estimates and downwardly biased standard errors; e.g., Sireci et al., 1991).

Finally, the concept of *measurement invariance* (e.g., Millsap, 2011) can be framed in terms of methods as well. Although invariance studies more commonly examine such things as the invariance of parameters across groups of persons (e.g., males versus females, persons of different nationalities), it is also possible to speak of invariance across alternative methods (commonly in terms of groups characterized by methods; e.g., persons who responded to a paper-and-pencil survey versus those who responded to an online version of the same survey). Here, concerns about methods may be worded in terms of construct bias, or the idea that a test may measure something different depending on its mode of delivery if group invariance does not hold. This hints at the possibility that methods may do more than simply introduce variance in observations over and above what can be attributed to variance in the measured attribute: they may, in fact, change the interpretation of the measured attribute.

Thus there is considerable variation in the conceptual vocabulary surrounding the concept of method-related dependencies in observations, and the motivations given for attending to such issues. This variation is intertwined with the semantics of the statistical techniques commonly employed in different fields to model method effects. These models and their semantics now deserve a closer inspection.

## The Semantics of Psychometric Models of Method Effects

Distinct methodological traditions have arisen in various areas of research in the human sciences. Included in these traditions are often strongly institutionalized preferences for particular sorts of statistical models. Although recent advances in generalized latent variable modeling (e.g., Skrondal and Rabe-Hesketh, 2004) have clarified the syntactic connections between commonly employed parametric models by establishing powerful unified frameworks from which these models can be derived as special cases, the vocabulary and traditions surrounding many specific models remain highly distinct, and the statistical and conceptual connections between various models are not readily transparent to the average researcher.

By way of illustration, two popular classes of models will be described here, with particular attention to both their formal semantics and the manners in which they are commonly interpreted: MTMM CFA models and random-effects IRT testlet models.

## Multi-trait Multi-Method CFA Models

After Campbell and Fiske’s (1959) introduction of the idea of an MTMM study and the rise of linear factor analysis (Lawley and Maxwell, 1963; Jöreskog, 1971; Bollen, 1989), CFA models were developed for MTMM data, the two most famous of which are the correlated-trait correlated-method (CTCM) model and the correlated uniqueness (CU) model (Marsh and Grayson, 1995). The CTCM model is commonly presented as follows:

(1) $$y_{j}=\tau +\Lambda_{T}T_{j}+\Lambda_{M}M_{j}+e_{j}$$

where **y***_j_* is a *p*-dimensional random vector of indicators (trait-method units) observed in subject *j* (*j* = 1 … *N*), **e***_j_* is a *p*-dimensional random vector of mutually uncorrelated residuals (also called specific factors, disturbances, or uniquenesses), τ is a *p*-dimensional vector of regression intercepts, **T***_j_* and **M***_j_* are *q*- and *r*-dimensional vectors of common Trait and Method factor scores, and Λ*_T_* and Λ*_M_* are the *p* × *q* and *p* × *r* matrices of trait and method factor loadings. Under the assumption that **T***_j_*, **M***_j_*, and **e***_j_* are mutually uncorrelated, the implied covariance structure is:

(2) $$\sum =\Lambda_{T}\Psi_{T}\overset{\prime }{\underset{T}{\Lambda }}+\Lambda_{M}\Psi_{M}\Lambda_{M}^{\prime }+\Theta$$

where Ψ*_T_* and Ψ*_M_* are the *q* × *q* and *r* × *r* covariance matrices of the trait and method factors and Θ is the diagonal covariance matrix of the residuals **e***_j_*.

**Figure 1:**
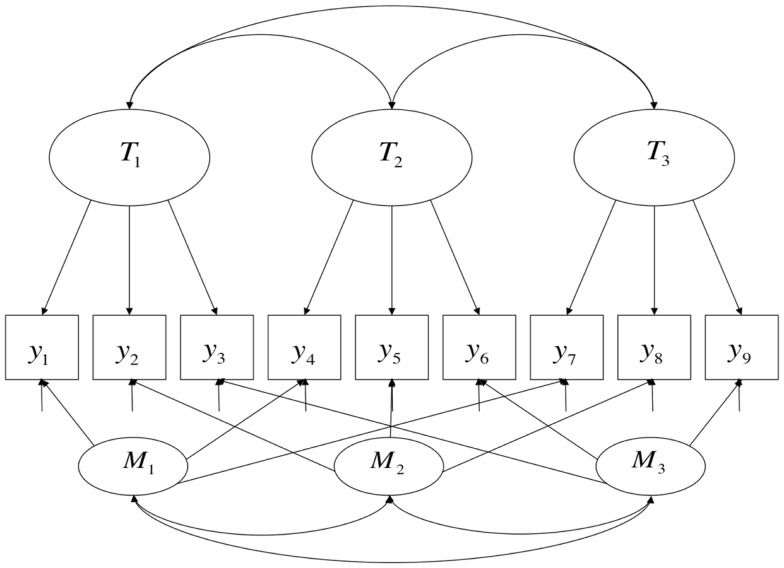
**A Correlated-Trait, Correlated-Method (CTCM) model**.

A path diagram corresponding to this model is shown in Figure 1 (here in the case in which there are three traits and three methods). This model, as a special case of linear confirmatory factor-analytic models more generally, models the conditional mean of the indicator variables, making it appropriate when said indicator variables are continuous. When estimated using maximum likelihood (ML) techniques (as is common), it must be additionally assumed that these variables are normally distributed. Since responses to individual test questions can rarely be scored continuously, item-level data are almost never modeled using CTCM models. Instead, it is common practice to take the sum or average of a set of items (the result is sometimes called a “parcel” or just a “test”), in the hopes that the distributions of such sums or averages approximate continuous and normally distributed variables. This model, again like factor models more generally, also assumes that the latent variables are continuous. Finally, MTMM studies nearly always have only a single indicator variable for each combination of trait and method, or “trait-method unit” (reflected in Figure 1 by the fact that there are 3M × 3T = 9 indicator variables), but there is no necessary reason this needs to be the case^3^; the number of trait factors times the number of method factors is simply the lower limit on the number of indicator variables needed for estimation of an MTMM.

Formally, the CTCM model states that population-level variance^4^ in each observed variable has three causes, each of which is modeled as a person-level random variable: the trait and method dimensions, and a specific factor. There are no formal semantic differences in the modeling of the trait and method causes other than their specific patterns of loadings (the fact that **T***_j_* and **M***_j_* are separated out in Eq. 1 is a simply a matter of notational convenience). In most applied contexts, the trait variables are interpreted in a relatively straightforward manner as denoting specified attributes (“traits”) of persons, controlling for the specific method of measurement. The method factors may be interpreted in a parallel manner as denoting attributes of persons that relate to how people respond to particular methods of measurement, controlling for which trait is being assessed, such as method-specific talents or skill sets; an example of such an interpretation would be when a factor loading on oral tests is interpreted as denoting “oral presentation ability,” controlling for the topic being spoken about. However, in practice, it is common for such dimensions to be interpreted primarily as nuisance dimensions, where only population-level parameters are of interest (similarly to how unique factors are commonly interpreted).

The fact that trait and method factors are both random person factors implies that term “method factor” may be considered misleading – that is, “method factors” vary over persons, not over methods. A somewhat more appropriate (albeit wordy) term might be “factors of method-specific ancillary traits.” The interpretation of these factors will be discussed further in a later section.

It is common for CTCM models to fail to converge, or to yield unstable or inadmissible solutions (Marsh and Bailey, 1991; Lance et al., 2002; Eid et al., 2008). Partly in reaction to this, modified forms of the CTCM model have been proposed, such as models with orthogonal methods factors correlated-trait uncorrelated-method (CTUM). The orthogonality constraints force a different set of semantics on the method factors; for example, the “oral presentation ability” interpretation from the previous example might be suspect, insofar as this ability might be expected to relate to facility with other methods, due, for example, to overlap in component skills (e.g., test-wiseness, motivation, general verbal ability). The method factor in the CTUM model could only be interpreted as denoting those attributes of persons that are truly specific to a particular method of measurement, and in many cases it may not be clear exactly what such attributes would be.

Another model proposed as an alternative to the CTCM model is the CU model (Marsh and Grayson, 1995), which drops the method factors entirely and allows the disturbances of observed variables that share a common method to correlate. Correlations are usually interpreted as “unanalyzed associations” in factor and path models, meaning that no causal explanation is given for these associations; thus, such models say nothing formal about the reasons methods induce dependence among observations.

## Item Response Theory Random-Effects Testlet Models

There are a number of within-item multidimensional IRT models that have been developed that could be considered models for method effects. As mentioned previously, the concept of LID shares a conceptual relation with the concept of method effects: LID occurs when variation in some subset of item responses shares more in common than just their common cause represented by the (primary) latent variable, and methodological similarities among items are an obvious possible source of such shared variance. Thus it could be said that method effects are one possible cause of LID, and, therefore, that models developed for LID may be used to model method effects.

**Figure 2:**
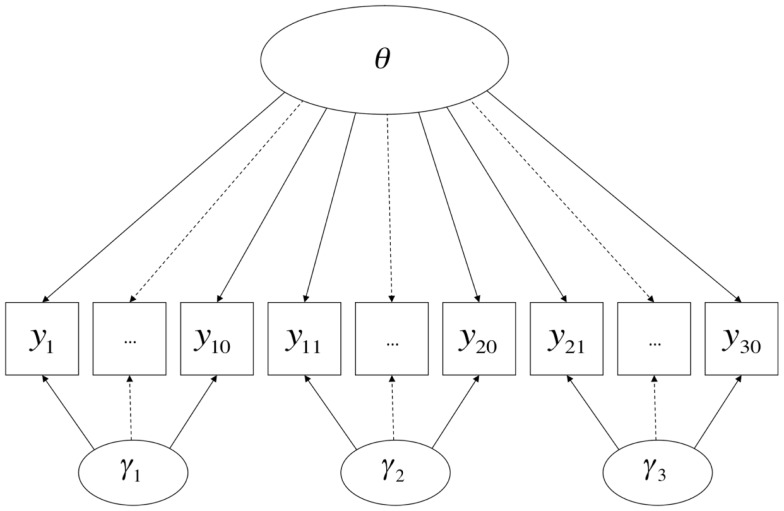
**A Bi-factor model**.

Various constrained versions of full information bi-factor models (Gibbons and Hedeker, 1992; see also Holzinger and Swineford, 1937) have been proposed to model tests with testlet structure, one of the more famous of which is Bradlow et al.’s, 1999; also see Wainer et al., 2007, c.f. Rijmen, 2010) testlet response model. The bi-factor model (and Bradlow et al.’s testlet model) can be pictorially represented by Figure 2 (here in the case in which there are three testlets and 30 items)^5^ Wang and Wilson (2005a,b) developed this model specifically for the one-parameter logistic (1PL) case. Their model is as follows:

(3) $$\log\left(P_{nij}/P_{ni\left(j-1\right)}\right)=\theta_{n}-d_{ij}-c_{k\left(i\right)}+\gamma_{nk\left(i\right)}$$

in which θ*_n_* represents the primary dimension of individual differences (also interpretable as a random intercept), *d_ij_* is the item parameter associated with step *j* in item *i*, *c_k(*i*)_* is the overall difficulty of testlet *k*, and γ*_nk(*i*)_* is the random effect of testlet *k*. The random-effects of testlets are constrained to be orthogonal to one another and to the random intercept θ*_n_*; thus, γ*_nk(*i*)_* can be interpreted as a dimension of individual differences (i.e., a random person effect) that affects the probability of success on items on a specific testlet, independent of the primary dimension and other testlets^6^. A separate equation for the covariance structure (analogous to Eq. 2) is not commonly given in the IRT literature (but would in this case be a diagonal matrix, given that all dimensions are orthogonal). The variances of θ*_n_* and γ*_nk(*i*)_* are estimated parameters and can be used to aid interpretation of the extent to which variation in item responses is attributable to variation in the primary dimension versus the presence of the item in its testlet. The formulation given here is consistent with Masters’ (1982) partial credit model, in which category difficulties are freely estimated for each item; appropriate constraints could yield other models (also see Adams et al., 1997).

This model can readily be extended to the 2PL case via the addition of item-specific slope parameters on θ*_n_* and γ*_nk_(*i*)*. In the case where slopes are estimated, there are various constraints that can be placed on the model for purposes of identification and interpretability: Bradlow et al. (1999) impose the restriction that a single slope parameter is estimated for each item, which applies to that item’s loading on both the primary dimension and the testlet dimension; other possibilities have also been explored, such as models that estimate a single slope parameter for each testlet dimension but allow all slopes to be freely estimated on the primary dimension (Li et al., 2006), and models that constrain the slopes of items on testlets to be inversely proportional to their slope on the primary dimension (DeMars, 2006).

The logit link function makes these models appropriate for ordinal indicator variables. The indicator variables are typically assumed to have Bernoulli distributions if dichotomous, and multinomial distributions if polytomous. As with all IRT models, it is assumed that the latent variables are continuous. Although the presentations of these models in the literature have assumed a single primary (“substantive”) dimension plus *k* testlet dimensions, they are easily generalizable to the case in which there is more than one substantive dimension, using established techniques in multidimensional IRT (e.g., Adams et al., 1997; Reckase, 2009), in which case the model would be multidimensional on both sides of Figure 2. If the items within a testlet loaded onto more than one substantive dimension, this model would be equivalent to the CTUM model discussed earlier, albeit with a logit rather than identity link.

As previously noted, these models have mainly been presented as “testlet” models and discussed in terms of their helpfulness in modeling specific sources of local dependence. Wang and Wilson (2005b) also refer to Eq. 3 as a “facet” model, and discuss how it can be applied when there are multiple raters whose influence cannot be modeled solely in terms of an overall shift in difficulty due to leniency or severity (i.e., a fixed effect, represented by *c_k_*), as is done in the many-facets Rasch model (Linacre, 1989). In terms of its formal semantics, the model is even more general than that. A random testlet or facet effect is equivalent to a second dimension of individual differences that causes variation in a subset of item responses, and thus, induces stronger dependence amongst those items than would be expected due to their primary common cause(s). There is no reason why the logic of this cannot extend to other sources of variation in specific subsets of items on a test, such as the fact that different subsets of items represent different sources of information (e.g., self-report, supervisor-report, etc.) or different formats of items, among other method-related possibilities.

Finally, like the CFA models discussed previously, the method (or testlet, or facet) dimensions vary over persons, not over methods; thus, again, a more appropriate name than (say) “testlet dimension” might again be “dimension of testlet-specific individual differences.” Conversely, the fixed effect *c_k_* is a constant over persons, and thus operates on the expectation (rather than the variance) of observed scores; taken together, a specific method can thus be modeled as adding both systematic bias and random error to the outcomes of measurement procedures.

## Discussion

The previous two sections have illustrated how the formal semantics of models for method effects depend on the particulars of model specifications (in terms of constraints, numbers of dimensions, choice of link functions, etc.). Informally, as discussed previously, interpretations of method effects depend largely on differences among research traditions in vocabulary, in the subject matter typically dealt with, and in the motivations commonly given for why method effects or LID are worthy of attention; these differences, combined with differences in the way the models are commonly presented (illustrated by the different choices of symbols and the switch between vector and scalar notation between Eqs 1 and 3, as well as the differences in baseline assumptions concerning the link function and the number of substantive dimensions in the model), may give rise to the perception that these models and their associated semantics are entirely dissimilar. This is, however, not the case: the models share a high degree of commonality at both the syntactic and semantic levels.

It is easier to see the connections between the models if one starts with a more general model, and then derives the earlier models. Using the notation of Skrondal and Rabe-Hesketh (2004), a generalized latent variable model can be formulated thusly:

(4) $$g\left({\text{y}}_{j}\right)={\text{X}}_{j}\beta +\Lambda_{j}\eta_{j}+\varepsilon_{j}$$

where **y***_j_* is a matrix of observed responses (which, among other possibilities, could be individual scored item responses, as is common in IRT, or parcel scores on tests, as is common in CFA), *g*(·) is a link function (e.g., identity, logit, probit, etc.), **X***_j_*β is the fixed part of the model, Λ*_j_* is a structure matrix describing the loadings of the latent variables η*_j_* onto the observed responses, and ε*_j_* is a vector of observation-specific errors or unique factors. This response model can be combined with a structural model:

(5) $$\eta_{j}=B\eta_{j}+\zeta_{j}$$

where **B** is an *M* by *M* matrix of regression parameters (modeling the covariance structure between the latent variables) and ζ*_j_* is a vector of *M* errors or unique factors.

Although this account leaves out many details, it summarizes the essence of a generalized latent variable model. From this model, the CTCM model (Eqs 1 and 2) can be derived by setting *g*(·) to an identity link, including no covariates in **X***_j_* and thus allowing β to be a vector of intercepts (denoted as τ in Eq. 1), splitting the notation for Λ*_j_*η*_j_* into a “trait” part and a “method” part (denoted as **L***_T_***T***_j_* and **L***_M_***M***_j_* in Eq. 1), and manipulating the contents of the **B** matrix to reflect the constraints on the factor correlations appropriate for the model. On the other hand, the random-effects Rasch testlet model (Eq. 3) can be derived from the generalized model by setting *g*(·) to an adjacent-category logit link, including covariates in **X***_j_* for the presence of an item in testlet *k* (with the value 1 for presence of an item in a testlet and 0 otherwise), splitting the notation for β into an item- (or item step-) specific intercept and a testlet-specific intercept (denoted as *d_ij_* and *c_k(*i*)_* in Eq. 3), splitting the notation for Λ*_j_*η*_j_* into a single primary dimension and *k* testlet dimensions (denoted as θ*_n_* and γ*_nk(*i*)_* in Eq. 3), and allowing **B** to be a diagonal matrix (thus constraining the primary dimension and all testlet dimensions to be mutually orthogonal), and constraining all elements of the Λ*_j_* matrix to be 1 or 0 (based on whether an item is or is not associated with each variable) rather than freely estimated. (Allowing the elements of the Λ*_j_* matrix to be estimated moves the model from the Rasch family to the 2PL family of IRT models).

Returning to a visual examination of Figures 1 and 2, it should be readily apparent that the two models share many features. Path diagrams such as these are traditionally silent as to the nature of the link function represented by the arrows – in classical CFA models, the arrows represent linear effects (i.e., identity links), but there is no necessary reason why they cannot represent non-linear effects – so the shift from identity links in Figure 1 to logit links in Figure 2 is not readily apparent. Also, the fixed part of the model (**X***_j_*β) is generally not represented in path diagrams, meaning that such diagrams are silent as to whether methods operate on the expectations of indicators in addition to their variances. These two omissions aside, however, it can be seen that in both cases variance in each indicator is influenced by two primary dimensions of individual differences, one of which is typically interpreted as denoting an attribute that the test was designed to measure, and the other of which denotes sources of variation associated with a particular method. Once one is aware that (a) there is no upper bound on the number of indicator variables that load onto each dimension and (b) there can be multiple substantive dimensions in IRT models just as in linear (CFA) models, it becomes clear that both models can easily be represented by the same path diagrams, with the exception of the absence of indicator-specific unique factors or error terms in the model with a non-linear link function.

Thus, perhaps despite appearances to the contrary, latent variable models employed in different research traditions share deep syntactic connections, and, accordingly, share much of their formal semantics as well.

## Theories of Measurement and the Concept of a Method Effect

In addition to the norms of statistical and interpretive practice associated with particular research traditions, thinking about method effects is also affected by beliefs (many of which may not be explicitly recognized by researchers) regarding the meaning of measurement itself. There is not a single consensus definition of measurement accepted by all human scientists, or indeed by all physical scientists, and debates over the meaning of measurement will likely not see resolution any time soon. Obviously, unclear semantics about measurement can propagate to unclear semantics about any measurement-related concept, including but not limited to method effects. It is worth reviewing some of the most influential lines of thought concerning measurement, and exploring how each of them has contributed to discourse on method effects, sometimes in contradictory ways. The various ways of thinking about measurement can broadly be categorized as either *empiricist* or *realist*.

### Empiricist thinking on measurement

The term *empiricism* can refer to a broad range of philosophical positions; they share in common a commitment to direct observation as the basis for knowledge (though what counts as *observation* is a perennially unsettled issue). Empiricism has been a major force in shaping Western thinking about science (and natural philosophy) since at least as far back as Aristotle, and standard accounts of the history of Western science emphasize how, over the centuries, empiricist lines of thinking have dovetailed with other views in epistemology (particularly those based on rationalism).

In the early twentieth century, the movement known as *logical positivism* synthesized many ideas from classical empiricism along with then-current advances in the philosophy of language and mathematics. Positivism was associated with a strong emphasis on direct observation as the basis for knowledge and a category rejection of metaphysics; statements regarding unobservable (theoretical) entities or forces were only regarded as meaningful if such statements could be linked to observations in a clear and consistent manner.

There are two major strands of thought on measurement that are consistent with much of positivist thinking. The first is *representational measurement theory* (RMT), which is characterized by the stance that measurement is the construction of morphisms between numerical relations and empirical relations (e.g., Scott and Suppes, 1958; Krantz et al., 1971; for an account of the relations between RMT and positivism, see Borsboom, 2005). The second is *operationalism*, which is characterized by the stance that the meaning of any theoretical concept is exhausted by the operations undertaken to measure instances of the concept (Bridgman, 1927). Both of these stances on measurement have exerted strong influences on thinking about measurement in psychology; notably, Stevens’ (1946) oft-cited definition of measurement as *the assignment of numerals to objects according to a rule* is consistent with both RMT and operationalism.

#### Representational measurement theory

Representationalism is regularly described as the mainstream position on measurement in the general literature the philosophy of science. It has also had a significant influence on thinking about measurement in the human sciences; however, with the exception of the relatively small body of literature in mathematical psychology from which the theory originated, most of this influence has been indirect.

Representational measurement theory holds that to measure is to construct a representation of an empirical relational system via a numerical relational system. On this view, the starting point for measurement is the determination of empirical relations amongst objects (e.g., X is greater than Y and less than Z). This requires that empirical relations be directly observable, or “identifiable” (Suppes and Zinnes, 1963, p. 7), though it is not always obvious what this means (c.f. Michell, 1990; Borsboom, 2005).

Once empirical relations are determined, numbers are assigned to empirical entities in such a way as to preserve the qualities of their empirical relations. Relational systems can possess different sorts of structures, and the particular sort of mapping of empirical onto numerical relations determines the scale properties. This is the basis for Stevens’ (1946) scale types (nominal, ordinal, interval, ratio), which are now common parlance in the human sciences. This is an example of the aforementioned indirect influence of RMT on thinking about measurement in the human sciences.

One of the principal reasons that RMT has not been more widely influential in the human sciences is that standard accounts of the theory have difficulty accounting for the role of measurement error. RMT holds that relations must be directly observable; in contrast, statistical models employed in the human sciences (such as those discussed in the previous section) take observations to be error-prone reflections of latent variables with idealized structures.

In fact, the very concept of a “method effect,” as formulated previously, piggybacks on the concept of error in observations. RMT’s requirement of observable relations would seem to require that the particular method of measurement employed play essentially no role at all, other than being a way of making such observations. One could formulate this hypothesis in (at least) two ways. In the first case, the method acts as a perfect conduit running from true relations in the world to sensed relations. In the second case, the concept of “true relations” is dismissed entirely, and sensed relations are themselves held to be the object of study. In this case, one could either hold that the world does not exist apart from our perceptions of it, or that its existence is simply irrelevant.

Where, then, do methods play a role in RMT? Without an account for how observations can contain error, it seems the only answer can be that either (a) the method of measurement plays a trivial role in being a perfect conduit from the real to the sensed world, or (b) the very concept of a method of measurement is unnecessary, as measurement is simply the mapping of directly experienced relations onto numerical relations. In either case, if two different methods of measurement yield two different relational systems, they cannot be said to be measuring the same attribute.

#### Operationalism

Operationalism (or operationism; Bridgman, 1927) shares with RMT a focus on observables as the basis of knowledge and a rejection of metaphysics. Operationalism was proposed as a semantic doctrine about the meaning of theoretical terms rather than a theory of measurement *per se*: operationalism holds that the meaning of theoretical terms is exhausted by the particular operations undertaken to observe them, which means that the results of a particular set of operations (or measurement procedure) are interpreted as measurements simply by fiat. Operationalism was originally proposed as a form of extreme epistemic humility in reaction to the upending of seemingly basic concepts such as length by the special theory of relativity: Bridgman felt that one of the reasons that it had been so difficult to see that the Newtonian notion of absolute time and space was flawed was that our theoretical terms came with too much baggage. For example, the idea of length was commonly interpreted as meaning something like “taking up space” – but this presupposes the existence of absolute space. Thus, asking why the lengths of objects seemed to be different depending on the speed with which they were traveling was already an ill-formed question, in that hidden within it was a false assumption about the nature of space. Bridgman proposed instead that the meaning of concepts such as “length” be limited to purely observable (operational) terms: thus “length” would mean nothing more than (for example) specified procedures associated with the repeated application of a standard meter stick.

Operationalism has since been almost uniformly rejected as irreconcilable with general scientific practice and vocabulary. Notably, operationalism has the consequence that each unique set of operations must be associated with a distinct theoretical term – thus, “the outcome of the application and reading of an alcohol thermometer” and “the outcome of the application and reading of a mercury thermometer” cannot refer to the same theoretical property. Following the collapse of logical positivism and an associated general retreat from extreme forms of empiricism, many scholars became increasingly willing to accept that the interpretation of concepts like temperature and intelligence outrun their associated measurement procedures – and, in fact, it is very difficult to make sense of both scientific and lay discourse about such concepts without this belief.

Operationalism had a strong influence on psychology (and in particular, behaviorism), especially through Boring (e.g., Boring, 1923) and his student Stevens (e.g., Stevens, 1935, 1946). One of the reasons for this is surely the difficulty of precisely defining psychological attributes; stating that “intelligence is what the tests test” (Boring, 1923, p. 35) neatly sidesteps the issue, and also gives at least the appearance of rigor by anchoring abstract ideas in observables. Notable in the concept of operationalism is that “it is meaningless to ask whether something is ‘really’ being measured … there is neither a need nor a place for postulating attributes which are prior to the measurement operation” (Borsboom, 2005, p. 93). Thus, just as in (at least one interpretation of) RMT, the very concept of a *method effect* is incoherent in an operationalist framework, insofar as method effects are held to “contribute variance to scores *beyond* what is attributable to the construct of interest” (Sechrest et al., 2000, emphasis added).

More generally, and again like RMT, the concept of measurement error is ill-fitting with operationalism: if the results of applying a procedure are by definition a measurement of the theoretical term, what is there to be in error about? If one were willing to accept that repeated applications of the same measurement procedure under the same conditions could yield different results, and one were willing to accept a definition of the theoretical term in terms of the average of a series of replications of a procedure rather than the outcome of a single application of that procedure, one could define measurement error as random deviations from a true long-run average; in fact, this is exactly how measurement error is defined in Classical Test Theory, a point argued by Borsboom (2005). As Borsboom also points out, repeated application of the same measurement procedure under the same conditions is not, strictly speaking, something that can ever be done, given that “the same conditions” must include the same time of measurement, and time moves in one direction only. Moreover, given our lack of access to the true counterfactual of running the same procedure under the same conditions, it is unclear why results should actually be expected to differ over identical applications. If results differ because the conditions are themselves different, then, according to the doctrine of operationalism, one does not have measurement error – one has distinct theoretical concepts.

Thus, at least in their original, strict forms, the two major lines of empiricist thought on measurement have little room for the concept of a method effect, as it is commonly interpreted in human science measurement. As soon as one has formulated the idea that an attribute of an object or person can be observed in more than one way, it seems one has also assigned an independent identity to the attribute, and embraced at least some version of a realist stance on measurement.

### Realist thinking on measurement

The term *realism* also refers to a broad range of positions; what they share in common is the belief that a natural world exists independently of observation. *Scientific realism* further proposes that at least one aim of science is to promote the acquisition of knowledge about this natural world. In the context of measurement (e.g., Michell, 2005) this implies that the measured attribute exists independently of the specific measurement procedure. It should be noted that while the strict forms of empiricism discussed in the previous section are either antirealist or simply arealist, there is nothing inherently contradictory about a commitment to observation as the basis of knowledge and the belief that a natural world exists independently of observation; thus, realist philosophies are often compatible with more moderated forms of empiricism.

There are various possible ways to conceive of the relationship between a measured attribute and the outcomes of a measurement procedure. Borsboom et al. (2003, 2004) and Borsboom (2005) articulate a *causal view of measurement*, which holds that measurement takes place when variation in an attribute causally produces variation in the outcomes of a measurement procedure, in such a way that inferences can be made from the outcomes of the procedure back to the attribute. For example, a mercury thermometer measures temperature because variation in temperature (the attribute) causally produces variation in the expansion of mercury in precisely calibrated glass tubes (the observations). The link of causality from the attribute to the outcomes of the procedure justifies the inference from those observed outcomes back to the unobserved attribute.

The validity of such a procedure is clearly threatened to the extent to which anything besides the targeted attribute can causally produce variation in the outcomes of the procedure. Broadly, this is consistent with Messick’s (1989) discussions of “construct-irrelevant variance.” One obvious source of such variance could be anything about the measured object that influences how it responds to a particular method of observation, apart from the fact that the method of observation is simply recording variation in the measured attribute. That is, if there is some other attribute of objects (e.g., their local air pressure) that influences how they respond to a particular measurement procedure (e.g., application of a mercury thermometer), then the extent to which variation in other such attributes is actually responsible for variation in the outcomes of the procedure could be considered a source of method-related attribute-irrelevant variance. Another source of such variance would be actual variance in methods, insofar each the outcomes of different methods applied to the same objects may have different expectations. It seems natural to apply the term “method effect” to both of these cases: that is, effects of methods that are random over persons, and effects that are fixed over persons.

Decoupling method-specific variance from attribute variance under a realist framework thus requires nothing more than knowing what attribute is the target of measurement, and how variance in this attribute is transmitted to variance in the outcomes of the measurement procedure. (Borsboom et al., 2009, p. 155 even state that this is “all there is to know regarding test validity”)^7^. If it is possible to give a complete account of the causal processes leading from variation in the attribute to variation in observations, any additional causes of variation in observations can be clearly identified as attribute-irrelevant, and threats to the validity of the measurement procedure. To the extent to which such additional sources of variation are associated with the particular method of observation used, they could be termed method effects. If the design of the test is thoughtful, models such as those discussed in the previous section may assist in purging (or “purifying”) person estimates of contamination by methods variance (cf., Ip, 2011).

This account raises an important conceptual point about method effects: inherent in the idea of a method effect is that, at least in principle, more than one measurement procedure (i.e., more than one method) could have been used to observe variation in the attribute; thus, the *particular* method by which variation in the attribute is observed in a given setting is attribute-irrelevant. In the case of human measurement, one interpretation of this phenomenon could be that people possess “ancillary skills” (Messick, 1996) that influence how well they perform on particular types of items or under particular conditions, and thus, how well a person does on any given test item is caused both by the attribute measured by the test and by skills unique to particular modes of assessment. This hypothesis is broadly consistent with the semantics of the MTMM and testlet models discussed earlier and displayed in Figures 1 and 2.

However, it may not always be clear to what extent an attribute is conceptually independent of the methods of measurement, especially in human science applications. The definition of temperature as an attribute of objects or systems is now very precise, and thermodynamic theory can specify the causal mechanisms that lead from variation in temperature to variation in the outcomes of the application of a range of specific measurement procedures (including but not limited to the aforementioned mercury thermometer) in a great amount of detail. Arguably, there are no cognitive theories so precisely developed, and the causal mechanisms that link attributes to observations are rarely if ever specified in such detail.

More generally, it is not always clear to what extent the method of observation is truly attribute-irrelevant, and to what extent the methods of observation help inform or even *construct* the meaning of the attribute. For example, consider an oral assessment of a student’s knowledge in a particular domain: it could be held that the attribute being assessed is simply knowledge in the domain, and the choice to assess it orally (as opposed to, for example, through multiple-choice items or written essays) is attribute-irrelevant; conversely, it could be held that what is being assessed is something more like “knowledge in the domain plus the ability to orally express that knowledge,” in which case the method of measurement is anything but irrelevant. Another example is a classic from the trait literature: it seems reasonable to suppose that a person may reliably behave differently in different social settings, such that their level of an attribute such as assertiveness truly differs when the person is with his or her family versus being at the workplace; in this instance a disparity between a family report and a supervisor-report of the person’s assertiveness may owe not to method-specific noise, but to a legitimate difference in the person’s context-specific manifestation of the trait. Though such interpretive difficulties have been acknowledged by a number of scholars, including Cronbach (e.g., Cronbach, 1995) and Campbell and Fiske themselves (e.g., Fiske and Campbell, 1992), lack of definitional clarity regarding what attributes are or are not intended to be measured by tests continues to obfuscate attempts to distinguish attribute-relevant from method-related-attribute-irrelevant variance in many applied contexts. In part, this may be because researchers are intuitively working from a metaphysical position that might be termed *constructive-realism* rather than a stricter form of realism that holds that attributes exist fully independently of human-designed measurement procedures.

#### Constructive-Realism

The concept of realism applied to psychological attributes is often taken to imply that the attributes in question are hypothesized to exist independently of human intentionality. That is, stating that an attribute *exists* or *is real* is taken to imply that it exists in observer-independent (ontologically objective) fashion, just like (supposedly) physical attributes such as temperature and mass. This, in turn, is often interpreted as implying physical (i.e., neurophysiological) identity, and in some contexts a genetically determined biological basis for variation in the attribute.

However, it is not necessary for psychological attributes to be ontologically objective for them to be real components of the natural world. Elsewhere, I have discussed how Searle’s (e.g., Searle, 1995) distinction between ontological and epistemic subjectivity and objectivity, and his recognition of the existence of intentionality-dependent objects and attributes of objects, provides the conceptual vocabulary with which a coherent realist account of psychological attributes can be formulated (Maul, 2012). Briefly, psychological attributes can be (a) to some extent ontologically subjective, in that they involve conscious phenomena with subjective first-person ontology, and (b) to some extent be composites delineated by contextually and pragmatically driven linguistic frames of reference, rather than being *natural kinds* (or natural attributes, as the case may be) in the classic sense (e.g., Quine, 1969). This is broadly compatible with Messick’s (1989) and Mislevy’s (e.g., Mislevy, 2009) *constructive-realism*, in which psychological attributes are taken to exist as reasoning devices in specific narrative frames of reference (see also Hood, 2009). Finally, this notion is compatible with many modern accounts of realism, such as Putnam’s (e.g., Putnam, 1999, p. 52) *natural* or *pragmatic realism*, which rejects the idea that the world consists of a fixed totality of mind-independent objects and their attributes and embraces the idea that “all our perceptions are already richly imbued with conceptual content … to use a Wittgensteinian idiom, seeing is always seeing as and it is the interface between the world and the rich fabric of our concepts that jointly determines what we see.”

**Figure 3:**
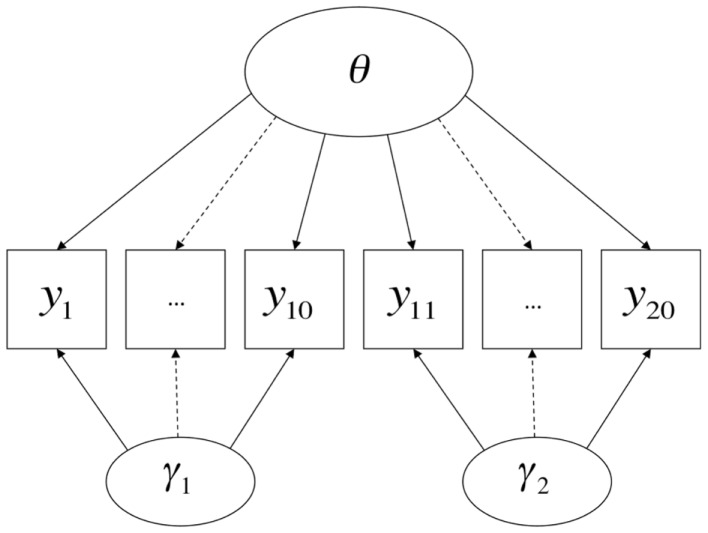
**Item types as method factors**.

**Figure 4:**
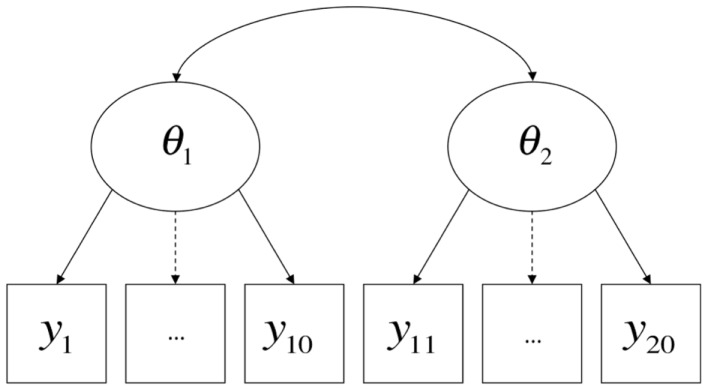
**Item types as primary dimensions**.

From this perspective, what constitutes a method effect is a contextualized and pragmatic issue, and methodological features of the very same procedure may be considered method effects or not relative to the conception of the attribute(s) being measured by the test. A contemporary example comes from the renewed interest on the part of the U.S. educational testing community in the inclusion of “performance events” (e.g., Messick, 1996) in high-stakes tests, alongside more traditional item formats such as multiple-choice [found, for example, in literature available from the Smarter Balanced Assessment Consortium (SBAC)^8^ and Partnership for Assessment of Readiness for College and Careers (PARCC)]^9^. On performance tasks, students may be asked to (for example) perform short experiments or produce specified products. Suppose that there is some degree of disparity between the results of performance events and multiple-choice items concerning the relative levels of knowledge of the students. It could be said that the two testing modalities each require a different set of method-specific ancillary skills in addition to the attribute intended to be measured (e.g., mastery of scientific concepts), and further that these ancillary skills are not themselves the target of instruction or measurement, in which case they are best treated as method factors (Figure 3); conversely, it could be argued that the two testing modalities measure legitimately and importantly different domains of knowledge, skill, and/or ability, in which case they are best treated as different substantive dimensions (Figure 4). Interestingly, much of the past and current rhetoric around the use of performance events in educational assessments is consistent with both possibilities (cf., Briggs and Maul, 2011).

**Figure 5:**
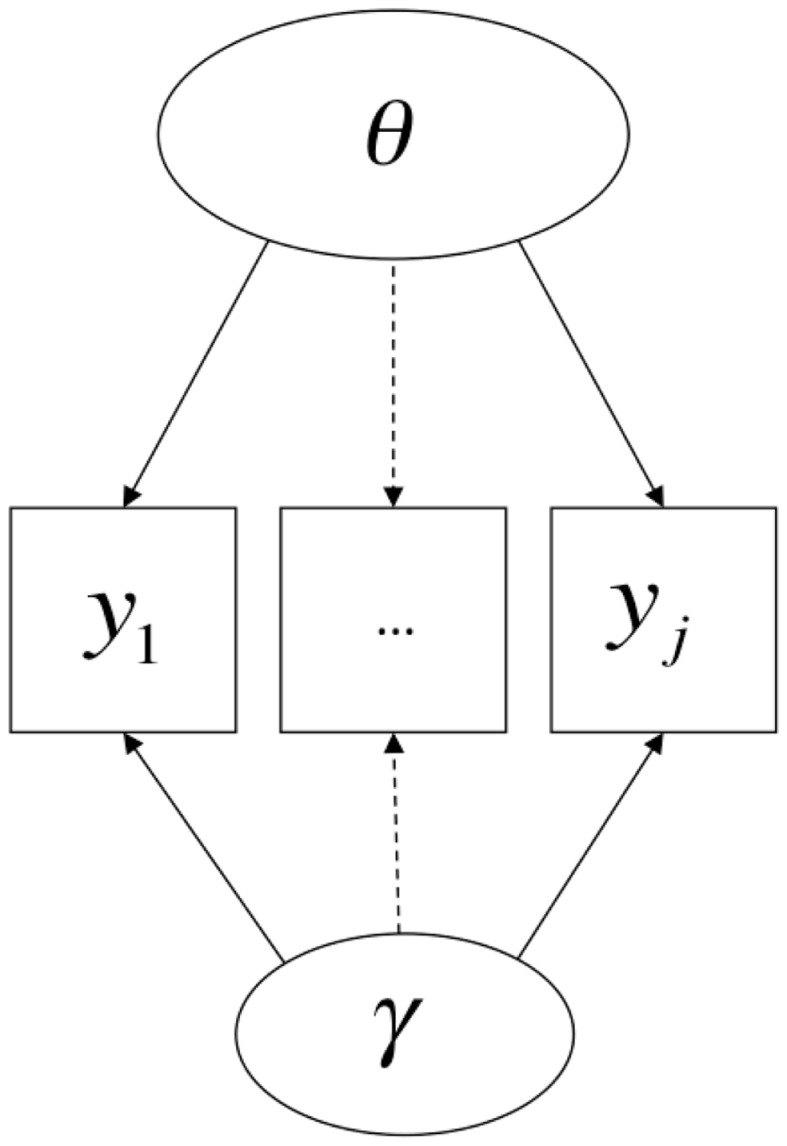
**Method confounded with trait**.

Still more troublingly, both possibilities could be true at once, in which case method-specific ancillary skills would be inseparable from attributes of interest. More generally, it may often be the case that a method of measurement is completely confounded with an intended target attribute. For example, any instance in which an attitudinal or motivational attribute is assessed entirely via positively worded Likert items is a situation in which each item response is potentially caused by both the attribute and by any ancillary person characteristics that influence how the person responds to positively worded Likert items (e.g., global agreeableness, edge aversion, or preference, idiosyncratic interpretation of the word “strongly,” etc.). This possibility is displayed visually in Figure 5. Under typical circumstances^10^, it will not be possible to estimate such a model; thus, a unidimensional model will likely be fit to the data, and it is likely that the unidimensional model will produce inflated estimates of the degree of dependence of the observations on the latent variable, insofar as there is an additional (method-related) source of dependence amongst the items being confounded with dependence due to the common causal attribute. Such situations are rarely discussed in terms of method effects, perhaps largely because they usually cannot be modeled as such, but the conceptual problem with method effects is very much present, and all the more intractable for being un-modelable.

Thus, in any given measurement situation, clarity regarding method effects depends on clarity regarding the ontological status of the attribute being measured. As was illustrated in the previous section, if an attribute is not taken to exist independently of a measurement procedure, the very concept of a method effect is incoherent; on the other hand, if it is taken to have independent existence, a coherent account of how the choice of methods influences the outcomes of the measurement procedure, and the selection of appropriate psychometric models, depends on being able to specify *a priori* what the attribute itself is and is not, and how the methods of measurement serve to transmit variance in the attribute into variance in observations.

## Discussion

Borsboom (2009, p. 708) commented that “semantics is … by far the weakest point of psychometric theory.” In this paper, I have argued that discourse and thinking around the concept of a method effect, and related ideas, are shaped by a number of sometimes-conflicting historical, normative, and philosophical forces, and that this has had the result of sometimes making it inordinately difficult to see the communalities across research traditions.

However, the formal semantics of models employed in different human science research traditions are in fact quite similar. Borsboom et al., 2003; borrowing terms from Hacking, 1983) discuss how latent variable models in general require both *entity realism*, in that they refer to unobserved attributes of persons or objects, and *theory realism*, in that they represent variation in the observed outcomes of measurement procedures as (at least in part) causally determined by variation in these attributes. The models discussed in this paper and presented in Figures 1–3 share these general features of latent variable models, and more specifically state that there are at least two causes of variation in specific observations, one of which is method-specific. Such a hypothesis may be compatible with a range of shades of realism, including versions of constructive-realism that allow for the possibility that the existence of an attribute is not independent of human intentionality. However, strictly interpreted antirealist theories, such as those derived from severe forms of empiricism popular in the early twentieth century, are not compatible with the concept of a method effect. If theoretical attributes were truly nothing more than the operations undertaken to measure them (operationalism), or if measurement were nothing more than the construction of morphisms from directly experienced empirical relations to numerical relations (representationalism), or indeed, if measurement were truly nothing more than the assignment of numerals to objects according to a rule (Stevens, 1946), method effects would be a non-issue. Measured attributes must exist independently of a specific measurement procedure if method effects are defined as sources of variance *beyond* what is attributable to the measured attribute.

Ultimately, method effects are but one of many specific measurement-related concepts in the human sciences that depend for coherence on clarity regarding deeper issues about the nature and meaning of measurement. The productivity and success of cross-disciplinary discourse (both among fields in the human sciences, and between the human and physical sciences) will depend on further clarification of many of the basic concepts of measurement.

## Conflict of Interest Statement

The authors declare that the research was conducted in the absence of any commercial or financial relationships that could be construed as a potential conflict of interest.

## Footnotes

^1^The language of convergent and discriminant validity was popular at the time largely because of Cronbach and Meehl’s (1955) seminal paper on construct validity. Although convergent and discriminant validity as such are not emphasized in modern treatments of validity (e.g., APA, AERA, NCME, 1999; Borsboom et al., 2004; Kane, 2006), their legacy remains present in the interpretations of MTMM studies, particularly in fields such as organizational and marketing research.

^2^Although multidimensional IRT models have now reached a fairly high state of sophistication, it is still more the norm of practice in the domains in which IRT is applied to consider only a single substantive dimension at a time, and therefore, it is much more common for LID to be modeled within a single (substantive) dimension rather than as a common source of item dependence across dimensions.

^3^In fact, models with more than one indicator at the intersection of each trait and method might be more resilient to the estimation and convergence problems so common in MTMM studies (for a review, see Lance et al., 2002).

^4^The semantics of models given in this paper correspond to what Borsboom et al. (2003) refer to as the between-subjects or interindividual interpretation of the causal relations expressed in latent variable models. As they discuss, when cross-sectional data are analyzed (as is common), this is the only interpretation that can be defended. Additional assumptions are necessary if a within-subjects or intraindividual interpretation is desired.

^5^Unlike the CFA literature, in the IRT literature it is uncommon to encounter path-diagrammatic presentations of models; to my knowledge the random-effects IRT testlet model has not been presented in this way before.

^6^As in the case of the CTUM model, this orthogonality constraint has the consequence that if there is any method- (or testlet-) induced variance in item scores that is *shared across methods* (or testlets), this variance will be confounded with the primary dimension rather than the method dimensions. In the case of a test composed of testlets, then, the variance of the testlet dimensions could be arguably interpreted as variance in responses due to factors specific to that very testlet, but not variance due to factors that would influence responses to testlet questions in general.

^7^Strictly speaking, one could argue that it is sufficient to know *that* variation in an attribute causes variation in observations, even if one did not know *how* this process occurred. In practice, it is difficult to know the former without knowing at least something about the latter.

^8^http://www.smarterbalanced.org/smarter-balanced-assessments/

^9^http://www.parcconline.org/parcc-assessment

^10^There are at least two circumstances under which such a model could be estimable. One is if it were possible to experimentally intervene on one dimension but not the other. Another is if it were possible to find subjects who could be assumed to have sufficiently high or low values on one of the dimensions such that there would be a ceiling or floor effect, and thus any variance in observed outcomes could be assumed to be due to variance in the other dimension.
